# The time is now: Preventing unnecessary criminalisation of children in care

**DOI:** 10.23889/ijpds.v8i2.2193

**Published:** 2023-09-14

**Authors:** Katie Hunter

**Affiliations:** 1 Manchester Metropolitan University, Manchester, United Kingdom

## Objectives

Newly linked administrative datasets allow us to explore the relationship between ethnicity, care experience and youth justice involvement. This data reveals huge disparities in the youth justice system, highlighting the need for urgent policy reform to prevent unnecessary criminalisation of children in care and care leavers.

## Method

Using linked administrative datasets from the Ministry of Justice and Department for Education, this paper takes a birth cohort approach to understanding youth justice involvement. It draws on National Pupil Database, Children Looked After and Police National Computer records from 2006 to 2017. Firstly, this paper will use descriptive statistics to highlight disparities in the extent of youth justice involvement among care-experienced individuals compared to non-care-experienced individuals and how this varies by ethnic group. Focusing on three offence types, the paper will then draw on mixed effects models to assess the likelihood of receiving a harsh sentence.

## Results

The paper will show that the extent of youth justice involvement among individuals who have been in care (i.e. foster care, children’s homes and kinship care) is much greater than previously thought. It will demonstrate that youth justice involvement is especially common among ethnic minority care-experienced individuals, particularly those who identify as Black or mixed ethnicity. The paper will highlight how care experience increases a person's risk of receiving a custodial sentence and may also affect their risk of getting a longer custodial sentence. Findings will be situated within the broader literature on in-care criminalisation (e.g. Walklate & Fitz-gibbon, 2021), calling attention to institutional and structural influences on justice systems involvement.

## Conclusion

The paper will show how linked administrative data can generate new insights which can inform policy and practice across both systems of care and justice. In doing so, it will make the case for a statutory duty of preventing unnecessary criminalisation of children in care and care leavers.

